# A novel hybrid deep learning approach combining deep feature attention and statistical validation for enhanced thyroid ultrasound segmentation

**DOI:** 10.1038/s41598-025-12602-6

**Published:** 2025-07-26

**Authors:** Tathagat Banerjee, Davinder Paul Singh, Debabrata Swain, Shubham Mahajan, Seifedine Kadry, Jungeun Kim

**Affiliations:** 1 Department of Computer Science and Engineering, IIT Patna, Patna, India; 2 Department of Computer Science and Engineering, Pandit Deendayal Energy University, Gandhinagar, Gujarat India; 3 Amity School of Engineering and Technology, Amity University Haryana, Gurugram, India; 4 Department of Computer Science and Mathematics, Lebanese American University, Beirut, Lebanon; 5 Department of Computer Engineering, Inha University, Incheon, Republic of South Korea

**Keywords:** Medical imaging, TATHA, Segmentation, Neural networks, Ultrasound imaging, Image processing, Engineering

## Abstract

An effective diagnosis system and suitable treatment planning require the precise segmentation of thyroid nodules in ultrasound imaging. The advancement of imaging technologies has not resolved traditional imaging challenges, which include noise issues, limited contrast, and dependency on operator choices, thus highlighting the need for automated, reliable solutions. The researchers developed TATHA, an innovative deep learning architecture dedicated to improving thyroid ultrasound image segmentation accuracy. The model is evaluated using the digital database of thyroid ultrasound images, which includes 99 cases across three subsets containing 134 labelled images for training, validation, and testing. It incorporates data pre-treatment procedures that reduce speckle noise and enhance contrast, while edge detection provides high-quality input for segmentation. TATHA outperforms U-Net, PSPNet, and Vision Transformers across various datasets and cross-validation folds, achieving superior Dice scores, accuracy, and AUC results. The distributed thyroid segmentation framework generates reliable predictions by combining results from multiple feature extraction units. The findings confirm that these advancements make TATHA an essential tool for clinicians and researchers in thyroid imaging and clinical applications.

## Introduction

The clinical disease known as thyroid nodules appears frequently among patients because these distinct lesions inside the thyroid gland can develop into benign or malignant conditions^1^. The prevalence of thyroid nodules has increased significantly because high-frequency ultrasound imaging diagnoses these nodules early and accurately. Patients with these nodules usually show no symptoms, yet specific ones might produce facial issues along with thyroid dysfunction and compression symptoms that require immediate medical care^2^. The visualization tests constitute the main diagnostic approach for thyroid nodules, but ultrasonography functions as their core methodology for assessment^3^. The diagnostic use of ultrasound produces fundamental data about nodule measurements, characteristics, and echogenicity properties, thus enabling professionals to detect benign from malignant tumors^4^. A sonographic evaluation is an effective tool for measuring nodule enlargement and collecting cytological material through fine-needle aspiration^5^. Ultrasound technology’s thyroid gland volume and nodule dimension assessment capabilities have increased due to technological advancements^6^. The problem of inconsistent ultrasound measurement findings between observers persists in medical practice ^7^. Nodule treatment traditionally included observation along with surgery and minor procedures or interventions. The potential treatment of benign thyroid nodules appears in radiofrequency ablation (RFA)^8^. As a minimally invasive technique, RFA uses thermal energy to kill affected cells, which reduces nodule size and improves symptoms and cosmetic appearance^9^. Research studies demonstrate that RFA safely and effectively addresses thyroid lesions, which makes it a rising choice over surgery for patients who cannot undergo surgery^10^. Multiple research studies analyze RFA’s technical aspects because interest in this procedure continues to rise^11^. Learning curve knowledge provides vital information for achieving reliable results while improving operator effectiveness^12^. Ultrasonography is a prevalent tool with RFA to monitor procedures actively and assess treatment results.

The precise diagnosis, together with treatment planning of thyroid nodules, depends heavily on ultrasound imaging segmentation because of its fundamental value in this field^13,14^. The exact implementation of segmentation technologies permits the measurement of thyroid volumetric data, which serves as fundamental information for both monitoring nodular growth and treatment planning^15–17^. The U-Net architecture stands as a prominent choice because it combines an encoder-decoder architecture to extract useful features while maintaining spatial awareness^18^. The 3D RoI-Aware U-Net stands as an advanced version of the U-Net architecture designed to improve medical imaging analysis performance for specific applications^19^. Cascaded CNNs serve the purpose of improved segmentation capabilities while refining outputs by progressively integrating high-level and low-level image elements^20^. According to research, ultrasonic picture segmentation models gain increased accuracy by integrating marker technology^21^. Modified U-Net (mU-Net) networks incorporate specific object characteristics that enhance their performance in complicated ultrasound dataset nodule detection and segmentation operations^22^. The established methods for multiple medical imaging applications, such as colorectal tumor and liver segmentation, continue to evolve for thyroid ultrasound imaging. ResNeSt split-attention networks alongside other models boost both feature extraction and model interpretability as explained in^23^. Metallic segmentation methods with atrous convolution technology have advanced the multiscale data management of medical pictures, which leads to better segmentation results^24^.

Frameworks such as RefineNet and encoder-decoder models utilizing atrous separable convolution have shown to be effective in enhancing segmentation outcomes by maintaining high-resolution details^25^. Extensive models employing deformable convolutions have demonstrated potential in tackling intricate medical imaging challenges, facilitating future progress in segmentation technology^26^. Deep learning frameworks like PyTorch have accelerated the swift creation and implementation of unique segmentation structures, offering a strong environment for testing and validation^27^. Techniques like ResNet and its derivatives have enhanced the domain by providing scalable and efficient solutions for deep learning-based picture segmentation^28^. These gains combined emphasize the substantial progress achieved in thyroid nodule segmentation, indicating the possibility for enhanced diagnostic accuracy and patient care^29^.

## Literature review

The progression of medical imaging methodologies has been crucial in enhancing diagnostic precision and therapeutic strategizing. Ultrasound imaging, due to its non-invasive nature, cost-effectiveness, and absence of radiation, has become an essential instrument in medical diagnostics, particularly for the evaluation of thyroid nodules. This study consolidates significant contributions from the literature on thyroid ultrasound imaging, segmentation, and associated deep learning methodologies, citing each reference in order. Orthogonal-plane convolutional networks have been shown to enhance needle identification in 3D ultrasound imaging, as evidenced by Pourtaherian et al.^30^. This method improves spatial comprehension and accuracy, establishing a basis for applications in other fields, including thyroid segmentation. Krönke et al.^31^ illustrated the efficacy of deep neural network-based thyroid segmentation in diminishing interobserver variability in thyroid volumetry, underscoring the significance of advanced models in clinical practice. Fedorov et al.^32^ made a substantial contribution to medical image computing by developing 3D Slicer, an open-source platform for quantitative imaging. This platform has significantly aided thyroid imaging studies by allowing the use of sophisticated segmentation methods. To tackle the widespread problem of speckle noise in ultrasound imaging, Yu and Acton^33^ proposed the speckle-reducing anisotropic diffusion approach, which maintains critical picture characteristics while diminishing noise, thus improving the quality of thyroid ultrasound images for segmentation. The U-Net design, developed by Ronneberger et al.^34^, represented a significant advancement in biomedical picture segmentation through the utilization of an encoder-decoder framework with skip connections. This design has been extensively utilized and adapted for thyroid segmentation tasks due to its superior precision in distinguishing anatomical components. Paszke et al.^35^ enhanced the deep learning community with PyTorch, an important and adaptable framework that has enabled the creation of intricate models for medical image analysis, including thyroid nodule segmentation.

The MONAI Consortium^36^ created MONAI, which delivers a devoted framework for medical imaging AI applications that provide tools and pre-trained models benefiting the creation of segmentation algorithms. A detailed analysis of thyroid segmentation methods appears in the work of Chen et al.^37^, where they discuss issues arising from noisy conditions and low visibility and operator dependency, but identify deep learning as an effective solution to overcome these challenges. Kumar et al.^38^ performed research on automatic thyroid nodule segmentation through deep learning, which showed that convolutional neural networks function well for reaching high accuracy and minimizing human intervention. The complex neural framework created by Ma et al.^39^ enables better recognition of thyroid glands together with surrounding tissues in ultrasound images, hence advancing segmentation model reliability. Active contour models used by Poudel et al.^40^ for thyroid ultrasound image three-dimensional segmentation provided confirmation of their success in creating precise border delineation. Poudel et al.^41^ conducted subsequent research to evaluate prevalent segmentation processes, where they emphasized the importance of machine learning to strengthen model performance in complex scenarios. Iommi et al. explained that 3D ultrasound delivers precise image guidance specifically during difficult medical procedures, as well as demonstrating its application potential for thyroid diagnosis^42^. Seifert et al.^43^ tested 3D ultrasound dataset integration to determine substantial thyroid volumes that would enhance volumetric analysis precision while improving thyroid evaluation performance. The research by Diarra^44^ described the optimization process of 2D matrix arrays for performing 3D ultrasound imaging while developing precise high-resolution imaging systems with better segmentation capabilities. Hatamizadeh et al.^45^ developed Swin UNETR as a transformer-based model for medical image semantic segmentation, as it improves performance in applications such as thyroid segmentation.

The researchers demonstrated the application of transformers through their work on UNETR to establish a whole framework for thyroid ultrasound data analysis^46^. Modern deep learning approaches have advanced the process of thyroid gland and nodule segmentation in ultrasound images. The authors of^47^ built upon the classical U-Net design to boost thyroid structure segmentation, thus showing how specific modifications work well for medical imaging tasks. The researchers from Mi et al.^48,49^ developed a method based on multi-scale feature fusion with hierarchical attention mechanisms to get better three-dimensional thyroid ultrasound image visualization outcomes for segmentation. The MLMSeg model presented by Chen et al.^50^ unites information across different ultrasound imaging angles for advancing thyroid nodule segmentation accuracy.

Biomedical image segmentation has witnessed significant advancements through the integration of attention mechanisms, optimization techniques, and deep learning frameworks. Recent studies have introduced models tailored for disease-specific segmentation and classification, such as ACE-SeizNet for seizure detection via EEG signals^51^, and IMATX for cervical cancer detection using interpretable AI^52^. Attention-guided architectures like DY-FSPAN^53^ and HHO-UNet-IAA^54^ demonstrate improved performance in histopathology and glaucoma segmentation tasks, respectively. Meanwhile, transfer learning and convolutional approaches, as seen in Falcon CNN for malaria detection^55^ and attention-based frameworks for pneumonia and Mycoplasma pneumoniae^56^, highlight robust diagnostic capabilities. Innovations also extend to deep belief networks^57^, GAN-augmented pneumonia diagnosis^58^, and multi-context organ segmentation models like Resio-Inception U-Net^59^. Recent efforts such as CICADA (UCX) further push the boundaries by enabling breast cancer aggressiveness delineation^60^, underscoring the growing role of automated segmentation systems in clinical decision-making.

### Highlights

- The process of thyroid nodule segmentation remains crucial for proper analysis and diagnosis, along with treatment design, followed by reducing subjective results in ultrasound imaging. Proper distinction between benign and malignant nodules is supported by this method.
- The study demonstrates deep learning methods U-Net and its advanced version TATHA provide superior feature extraction together with segmenting precision in thyroid ultrasound image analysis.
- The Tri Attribute Hybrid Algorithm (TATHA) achieves superior segmentation results because it applies attention mechanisms and dilated convolutions together with skip connections into its advanced design.
- TATHA delivers superior performance compared to U-Net, PSPNet, and ViT while serving different datasets, as it achieves better segmentation results through high accuracy and Dice scores as well as AUC values.
- The Distributed Thyroid Segmentation Framework (DTSF) utilizes a distributed modeling system to assure reliable segmentation by integrating response output from multiple feature extraction units, achieving superior generalization and variational data control.

## Methodology

The segmentation of thyroid ultrasound is of extreme importance in current medical imaging and diagnosis, particularly in diagnosing thyroid gland abnormalities. The affected part of the body is the thyroid, which is a small gland situated in the neck, just below the Adam’s Apple. Benign diseases, including thyroid nodules, goiters, and malignant diseases, can be visualized through ultrasound because that technique is safe, cost-effective, and devoid of radiation. Nonetheless, the analysis of ultrasound images using conventional methods is difficult because of noise, low contrast, and its operator dependency. This is where thyroid ultrasound segmentation becomes useful or rather vital in this case.

Morphological analysis encompasses the ability to separate the thyroid gland or specific areas of interest, such as nodules, from other surrounding tissues so that objective and specific quantitative measurements can be made. Segmentation provides better accuracy for recognizing important aspects of nodule morphology, including boundaries, sizes, and internal structures, when this process is being automated. The correct segmentation is especially important for thyroid nodules’ classification as benign or malignant, as small differences in the texture, shape, and echogenicity can be significant features. These conclusions help clinicians isolate the need for further procedures, including fine-needle aspiration biopsy. The proposed thyroid ultrasound segmentation reduces inter-operator variability, which is frequently encountered in manual analysis. These guides make it easier for anyone to identify regions of interest, and when used consistently, they increase diagnostic reproducibility across practitioners and institutions. This is particularly important in big population-based epidemiological investigations or clinical research studies performed across multiple centres where stringency in data analysis is paramount. In recent years, it has also extended as a fundamental paradigm of sophisticated computational methodologies in thyroid imaging. Deep learning and machine learning algorithms require an accurately segmented dataset of images for either their training or for validation. Researchers have used convolutional neural networks and U-Net-based structures for the segmentation of thyroid UK images in an automated manner. In addition to the speed and quality of segmentation, they can be easily incorporated into vast diagnostic systems, providing real-time, fully automated processes.

Moreover, precise segmentation enables a volumetric assessment, which becomes particularly important when studying the dynamics of nodules’ growth or thyroid volume. This has important consequences for the activity of judging disease progression, treatment efficacy, and inducing signs of relapse during follow-up examinations. Some of the explanations are: In cases of thyroid cancer, accurate segmentation helps us to plan our surgical approaches in terms of the facial boundary of the gland and its association with its neighbours. Besides, thyroid ultrasound segmentation serves in clinical practice, and it also provides its input in research activities. Segmentation techniques, hence, offer a structure in the design of new algorithms and enhancement of imaging procedures. These steps are informed by knowledge deduced from such research and help fashion novel techniques in both diagnosing the disease and treating the affected.

### Data description

The digital database of thyroid ultrasound images (DDTI) is an open-access resource aimed at supporting research in the development of Computer-Aided Diagnosis (CAD) systems for the analysis of thyroid nodules. It is designed to serve as both a training and teaching tool for radiologists and researchers in the field. The primary goal of this dataset is to facilitate the creation of algorithms that can assist in the diagnosis and evaluation of thyroid conditions.

Table 1 Training, Testing, and Validation Cases provides the overall distribution of images into training, validation, and test sets for a single run of training. Table 2 Cross-Validation Fold Sets for Testing details the cross-validation setup across 15 folds, ensuring that the model is evaluated in a more comprehensive manner by using different subsets of the data for testing in each fold. Both tables together provide a clear and structured approach to data splitting for reliable model evaluation. Both Tables 1 and 2 represent the same dataset derived from the DDTI database, following a holdout strategy with a 70:15:14 ratio for training, validation, and testing. A minor correction has been made to Table 1, where the number of training images is now accurately reported as 450, and the number of test images as 92. Table 1 reflects the static split used during early experimentation, while Table 2 illustrates a cross-validation setup based on the same data distribution. In each of the 15 folds, the number of training images is fixed at 450, and the validation and test sets are varied using random sampling to ensure diversity and generalizability. This approach enables more comprehensive evaluation, and the final results reported are derived from this 15-fold configuration.

**Table 1:** Training, testing, and validation cases.

Set type	Number of cases	Number of images
Training set	70	450
Validation set	15	95
Test set	14	92

**Table 2:** Cross-validation fold sets for testing.

Fold	Training set (images)	Validation set (images)	Test set (images)
FOLD 1	450	95	92
FOLD 2	450	128	92
FOLD 3	450	106	92
FOLD 4	450	120	92
FOLD 5	450	147	92
FOLD 6	450	133	92
FOLD 7	450	115	92
FOLD 8	450	100	92
FOLD 9	450	105	92
FOLD 10	450	117	92
FOLD 11	450	110	92
FOLD 12	450	122	92
FOLD 13	450	130	92
FOLD 14	450	93	92
FOLD 15	450	125	92

### Data preprocessing

#### Speckle noise reduction using adaptive filters

The reflection wave interferences during ultrasound imaging produce speckle noise artifacts in image results. Bilateral filtering stands apart from adaptive filters, such as median filters and anisotropic diffusion filters, when it comes to noise suppression processes. These filters maintain crucial edges, together with their ability to remove random noise, which allows essential features like nodule boundaries to stay free from blurring. The updated pixel intensity under Eq. 1 represents the noise-reduced pixel intensity value, which evolves through iterative calculations. The image-smoothing process is adjusted according to the value set in the conditioning parameter. A higher value gives excessive edge blurring, yet setting it too low allows excessive noise retention. The perpendicular relationship between adjacent pixels generates spatial gradients that measure the intensity difference between them. Gradients provide edge detection capabilities because they show significant changes between pixels. The amount of smoothing that takes place in the image depends on the value of the conduction coefficient and the gradient magnitude. The process smooths strong edges approximately the least amount, but provides increased smoothing to relatively flat sections to keep crucial structures unaltered.

1 $$I\left( {x,y,t + 1} \right) = I\left( {x,y,t} \right) + {\uplambda }\mathop \sum \limits_{i = 1}^{4} c_{i} \cdot \nabla I_{i}$$

#### Contrast enhancement using histogram equalization

The low image contrast levels in thyroid ultrasound examinations make it challenging for medical professionals to properly separate the gland and nodules from adjacent tissues. The range of brightness values in images is determined by intensity levels, which are specified in Eqs. 2 and 3 during contrast enhancement using CLAHE. Size calculation for re-distributing intensities happens by assessing the overall number of image pixels. The frequency distribution of image intensities appears in the histogram count, which defines the transformation function and other parameters.

2 $$T\left( r \right) = \frac{L - 1}{{MN}}\mathop \sum \limits_{i = 0}^{r} h\left( i \right)$$

3 $$T_{{{\text{CLAHE}}}} \left( r \right) = \min \left( {T\left( r \right),T_{{{\text{clip}}}} } \right)$$

#### Edge detection for boundary refinement

Correct identification of thyroid gland borders and nodules must be precise for proper segmentation processes. Significant edges in an image become visible with the help of detection methods, including the Canny Edge Detector and the Sobel operators. The techniques establish distinct boundaries between thyroid tissue and its adjacent structures, thus minimizing segmentation-related mistakes. Edge refinement methods find specific benefits when working with the problems that result from indistinct or unclear nodular borders in ultrasound images. The edge detection process depends on Eqs. 3 and 4 Calculate the gradient magnitude through measurements of image intensity change, which detects significant edges. The directional information from edge orientation indicates how the intensity changes to enable the identification of horizontal, vertical, or diagonal edges. The thresholding technique divides edges into meaningful boundaries as well as trivial boundaries for classification purposes. Two threshold points work together: the first detects robust edges at a high level, while the second joins weaker edge segments to stronger ones. The result of this operation leads to accurate boundary recognition along with the prevention of excess noise detection.

4 $$G\left( {x,y} \right) = \sqrt {\left( {\frac{\partial I}{{\partial x}}} \right)^{2} + \left( {\frac{\partial I}{{\partial y}}} \right)^{2} }$$

5 $${\uptheta }\left( {x,y} \right) = \arctan \left( {\frac{{\frac{\partial I}{{\partial y}}}}{{\frac{\partial I}{{\partial x}}}}} \right)$$

#### Region of interest (ROI) extraction

The visual content of thyroid ultrasound examinations frequently contains non-relevant parts that consist of neck tissue area or outside artifacts beyond the thyroid boundaries. When ROI extraction operates on thyroid gland segments, it decreases both computational complexity and increases overall accuracy rates. This process employs methods that include template matching, together with automatic bounding box generation, which depends on initial detection results. The accuracy of ROI extraction helps the segmentation algorithm to perform analysis on important data points alone, which avoids distractions from extraneous regions. For region of interest extraction, the bounding box parameters specify the rectangular boundaries that encompass thyroid gland or nodule regions according to Eq. 6. The rectangle edges defined by minimum and maximum coordinates specify the area of interest for analysis. Through this step, the segmentation algorithm operates solely on necessary regions because it removes all superfluous areas, including adjacent tissues or imaging artifacts.

6 $$B = \left\{ {\left( {x,y} \right)|x_{\min } \le x \le x_{\max } ,\;y_{\min } \le y \le y_{\max } } \right\}$$

### Need for distributed modelling for thyroid segmentation

The requirements for distributed modelling in thyroid ultrasound segmentation stem from two main problems, which include the extraction bottlenecks affecting features and the high GPU processing needs. A system for thyroid segmentation features two main operational modules that overcome existing system obstacles effectively.

The initial aspect utilizes Thyroid Feature Extraction Network (ThyroFEN) as a specialized ultrasound thyroid image processing scheme dedicated to feature extraction. The network applies its algorithms to spot essential image areas in ultrasound elements before mapping probabilities that determine pixel space membership for background sections and thyroid region elements.

**Fig. 1:**
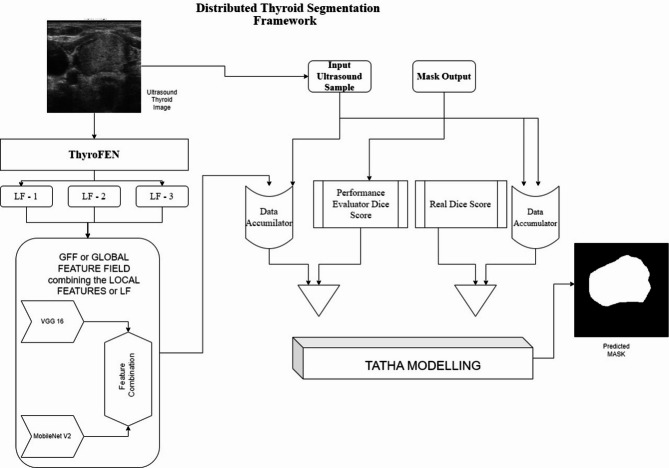
Distributed thyroid segmentation framework.

DTSF functions (Fig. 1) as the second component by integrating various feature extraction units through which trained weights determine output significance. A distributed framework ensures the model depends on multiple extractor units because DTSF utilizes various models in an ensemble configuration to perform precise medical image segmentation. DTSF operates as an integration system that handles the varying outputs of individual feature extractors to accomplish trustworthy predictions, especially for new data.

#### ThyroFEN

The Thyroid Feature Extraction Network (ThyroFEN) plays a pivotal role in thyroid segmentation by focusing on localized feature extraction. Local feature extraction serves as the central function of the Thyroid Feature Extraction Network (ThyroFEN) during thyroid segmentation applications. The network accomplishes two functions: building an attention framework that detects useful thyroid gland patterns and maintaining stable segmentation results across varying local conditions. ThyroFEN achieves this functionality through its combination of U-Net with an Inception Bi-residual backbone and VGG16 and MobileNet V2 architectural components, which are specified through Eq. 7. VGG16 extracts discriminant image features from ultrasound scans, yet MobileNet V2 provides an efficient platform for processing tasks without sacrificing performance outcomes. The network obtains detailed information about thyroid gland structures, including nodules and texture patterns, using this backbone structure, which reduces its vulnerability to specific patient-related features. The network design’s robust capabilities produce reliable features that allow high consistency in processing different patients and datasets before moving forward to the Distributed Thyroid Segmentation Framework (DTSF) distributed processes.

7 $${\text{ThyroFEN}}_{i} = f_{{{\text{extract}}}} \left( {I_{i} ,{\uptheta }_{{{\text{ThyroFEN}}}} } \right)$$

#### Distributed Thyroid Segmentation Framework (DTSF)

The DTSF module deals with problems arising from different local feature extractors, particularly during ultrasound thyroid image analysis. The system’s distributed architecture maintains segmentation independent from individual models, achieving better data generalization for unknown data. The DTSF addresses necessary questions connected to choosing the most trustworthy output from multiple ThyroFEN units through Eq. 8 while examining the benefits of distributed architecture and methods to handle segmentation conflicts between masked and unmasked areas. The distributed approach deployed by the DTSF enables merging different local feature extractor outputs into a single unified segmentation.

8 $${\text{DTSF}}_{{{\text{output}}}} = \mathop \sum \limits_{i = 1}^{N} w_{i} \cdot {\text{ThyroFEN}}_{i}$$

### Tri-attribute hybrid algorithm or TATHA

The Tri Attribute Hybrid Algorithm architecture, or TATHA, functions as a specialized deep learning model exclusively for image segmentation applications, which optimizes the processing of local contextual information and global contextual information through its implemented T-blocks. TATHA uses T-blocks that unite CNN techniques with contemporary attention methods across successful designs, which include U-Net, V-Net, and Vision Transformers (ViTs). TATHA successfully integrates abilities to handle lower and higher-level image characteristics during processing, thus making it an ideal solution for medical image segmentation where precise localization needs and high accuracy demands are critical. Table 3 Architecture and blocks deploys regular and dilated convolutions to obtain multi-scale features. Through dilated convolutions, the receptive field grows, making the network able to analyze broader areas of context without losing image detail clarity. Complex spatial tasks especially require this technology for segmenting medical images, such as organs or tumors. The channel attention modules within TATHA dynamically manage feature importance at different channels. The adaptive feature selection capability of the network directs it to attend to pertinent characteristics that ultimately boost its segmentation outcome.

**Table 3:** Architecture and blocks.

Module	Input	Output	Operations
T-Block (dilated and regular convolutions)	Feature map x	Processed feature map out	1. Apply standard 3 × 3 convolution to x
			2. Apply dilated 3 × 3 convolution to x
			3. Concatenate both results
			4. Apply Batch Normalization and ReLU activation
Channel attention module	Feature map x	Attended feature map out	1. Compute global average pooling and global max pooling of x
			2. Pass pooled results through shared dense layers
			3. Combine average and max pooling outputs with addition
			4. Apply sigmoid activation and multiply input x with attention map
Decoder block with skip connections	Current feature map x, Skip features from encoder	Refined feature map x	1. Apply transposed convolution (upsampling) to x
			2. Concatenate upsampled x with skip connection from encoder
			3. Apply T-block to processed features
T-Net (encoder–decoder architecture)	Input image inputs	Segmented output	1. Encoder: Apply T-blocks with pooling layers, and optionally add VGG16 backbone
			2. Bottleneck: Apply T-block and channel attention
			3. Decoder: Apply decoder blocks with skip connections from encoder
			4. Final output: Apply 1 × 1 convolution for segmentation
Attention map visualization	Model, input image input_image	Attention map	1. Define intermediate model output at attention layer
			2. Predict attention map by passing input through this intermediate model

Another key feature of TATHA is its use of skip connections and up sampling layers showcased by Table 4 T Net Architecture in the decoder part of the architecture. These connections help preserve high-resolution spatial information from earlier layers, which is crucial for accurate pixel-wise classification. By using a combination of dilated convolutions, attention mechanisms, and skip connections, TATHA is able to capture both detailed and global context, making it highly effective for complex segmentation tasks.

**Table 4:** T net architecture.

Layer	Output shape	Parameters	Connections
Input	(None, 256, 256, 1)	0	–
Conv1	(None, 256, 256, 64)	640	Input
Conv2	(None, 256, 256, 64)	640	Conv1
Concat1	(None, 256, 256, 128)	0	Conv1, Conv2
BN1	(None, 256, 256, 128)	512	Concat1
ReLU1	(None, 256, 256, 128)	0	BN1
MaxPool1	(None, 128, 128, 128)	0	ReLU1
Conv3	(None, 128, 128, 128)	147,584	MaxPool1
Conv4	(None, 128, 128, 128)	147,584	Conv3
Concat2	(None, 128, 128, 256)	0	Conv3, Conv4
BN2	(None, 128, 128, 256)	1024	Concat2
ReLU2	(None, 128, 128, 256)	0	BN2
MaxPool2	(None, 64, 64, 256)	0	ReLU2
Conv5	(None, 64, 64, 256)	590,080	MaxPool2
Conv6	(None, 64, 64, 256)	590,080	Conv5
Concat3	(None, 64, 64, 512)	0	Conv5, Conv6
BN3	(None, 64, 64, 512)	2048	Concat3
ReLU3	(None, 64, 64, 512)	0	BN3
MaxPool3	(None, 32, 32, 512)	0	ReLU3
Conv7	(None, 32, 32, 512)	2,359,808	MaxPool3
Conv8	(None, 32, 32, 512)	2,359,808	Conv7
Concat4	(None, 32, 32, 1024)	0	Conv7, Conv8
BN4	(None, 32, 32, 1024)	4096	Concat4
ReLU4	(None, 32, 32, 1024)	0	BN4
MaxPool4	(None, 16, 16, 1024)	0	ReLU4
Conv9	(None, 16, 16, 1024)	9,438,208	MaxPool4
Conv10	(None, 16, 16, 1024)	9,438,208	Conv9
Concat5	(None, 16, 16, 2048)	0	Conv9, Conv10
BN5	(None, 16, 16, 2048)	8192	Concat5
ReLU5	(None, 16, 16, 2048)	0	BN5
GlobalAvgPool	(None, 2048)	0	ReLU5
GlobalMaxPool	(None, 2048)	0	ReLU5
Dense	(None, 2048)	524,544	GlobalAvgPool, GlobalMaxPool
Add	(None, 2048)	0	Dense
Mul	(None, 16, 16, 2048)	0	ReLU5, Add
UpConv1	(None, 32, 32, 512)	9,437,696	Mul
Concat6	(None, 32, 32, 1536)	0	UpConv1, ReLU4
Conv11	(None, 32, 32, 512)	7,078,400	Concat6
Conv12	(None, 32, 32, 512)	7,078,400	Concat6
Concat7	(None, 32, 32, 1024)	0	Conv11, Conv12
BN6	(None, 32, 32, 1024)	4096	Concat7
ReLU6	(None, 32, 32, 1024)	0	BN6
UpConv2	(None, 64, 64, 256)	2,359,552	ReLU6
Concat8	(None, 64, 64, 768)	0	UpConv2, ReLU3
Conv13	(None, 64, 64, 256)	1,769,728	Concat8
Conv14	(None, 64, 64, 256)	1,769,728	Concat8
Output	(None, 64, 64, 1)	0	Conv14

#### Architectural benefits from other competitive segmentation frameworks

##### UNET and VNET

The TATHA architecture draws significant inspiration from U-Net, particularly its encoder-decoder structure and the use of skip connections. U-Net is known for its ability to generate high-quality segmentation maps, especially for biomedical image segmentation, by utilizing its skip connections to transmit low-level features from the encoder to the decoder. This feature is preserved in T-Net, which also benefits from the effective preservation of fine details during the decoding phase.

##### Vision transformers (ViT)

The integration of Vision Transformers (ViTs) into TATHA introduces the concept of global context modelling. ViTs have shown impressive results in image classification and segmentation by leveraging self-attention mechanisms to model long-range dependencies across the entire image. In T-Net, the addition of attention modules (similar to ViTs) allows the network to focus on relevant regions of the image dynamically, which is particularly beneficial when dealing with large images where distant spatial dependencies are crucial. The global context captured through self-attention helps TATHA better segment objects that may be distant or have complex spatial relationships with other structures in the image. This improves the model’s ability to deal with varying image qualities and resolutions, a challenge often encountered in real-world image segmentation tasks.

#### Banerjee coefficient for loss

Banerjee Coefficient for Loss, as shown in Eq. 9, is a blend of two functions to handle specific problems of segmentations in ultrasound images, that is, the thyroid segmentation in this study. This loss function is semi-automated and achieves the appropriate boundary delineation within smaller and more complex and irregular shapes, including the thyroid gland in the presence of background tissues. Thyroid images utilised by ultrasound have issues such as noise and artefacts, and unbalanced datasets, and hence pose a great challenge when segmenting them. The Banerjee Coefficient Loss eliminates a number of weaknesses presented by older segmentation loss functions; the ability to adjust the false positive/false negative ratio dynamically allows for increased accuracy in the segmentation process. This must be done in clinical settings, for two possible reasons: over-segmentation may produce false positives, and under-segmentation may produce false negatives. Furthermore, the loss function refines the intersection of the predicted regions with the ground truth regions by improving shape and size understanding of the thyroid gland. This feature is very useful when tracing organs that lack clear margins or that are overlaid by artefacts, as is typically the case in ultrasound studies. These challenges listed above are effectively handled by the Banerjee Coefficient Loss to make the segmentation reliable and precise in every picture quality, patient position, and scanning angle. Finally, this novel loss function benefits from upgrading the model performance and is ideal for clinical applications that require high-quality and accurate segmentation, which would help in diagnosis and implementation of initial treatment plans.

9 $$\begin{gathered} L_{{\text{Banerjee Coeff}}} = 0.23 \cdot \left( {1 - \frac{{\left| {P \cap G} \right|}}{{\left| {P \cap G} \right| + {\upalpha }\left| {P - G} \right| + {\upbeta }\left| {G - P} \right|}}} \right) \hfill \\ + 0.65 \cdot \left( {1 - \frac{{2\left| {P \cap G} \right|}}{\left| P \right| + \left| G \right|}} \right) + 0.12 \cdot \left( {\left( {1 - \frac{{\left| {P \cap G} \right|}}{{\left| {P \cap G} \right| + {\upalpha }\left| {P - G} \right| + {\upbeta }\left| {G - P} \right|}}} \right)^{{\upgamma }} } \right) \hfill \\ \end{gathered}$$

#### Hyperparameter for T Net

The hyperparameter table, in Table 5, for the TATHA architecture outlines key configuration settings that are essential for building a robust model for medical image segmentation tasks. The model uses an input shape of (256, 256, 1), designed for grayscale images, which is commonly used for applications like thyroid nodule detection. The T-blocks, a core component of the encoder path, progressively use filters of increasing sizes (64, 128, 256, 512, 1024) to capture both fine and high-level features, with a dilation rate of 2 applied to dilated convolutions to enhance the receptive field. ReLU is used as the activation function across convolutional layers, enabling the model to introduce non-linearity and accelerate training.

**Table 5:** Hyperparameter table for T net.

Hyperparameter	Value
Input shape	(256, 256, 1)
Filters (T-block)	64, 128, 256, 512, 1024
Dilation rate (T-block)	2
Activation (Conv layers)	‘relu’
Pooling size (MaxPooling2D)	(2, 2)
Batch normalization	TRUE
Attention ratio (Channel attention)	8
Optimizer	‘adam’
Loss Function	Banerjee’s Coefficient
Metrics	[‘accuracy’]
Num classes (Output)	1
Backbone model	None
Conv2D Kernel size	(3, 3)
Conv2DTranspose Kernel size	(3, 3)
Dropout (if used)	Not specified
Epochs	20–100
Batch size	16–32

## Results and discussion

The section corresponds to multiple experiments conducted in this study, serving as a comprehensive evaluation of the proposed model across various facets of its performance, robustness, and comparison with existing methods.

### Experiment 1: dataset preview

**Fig. 2:**
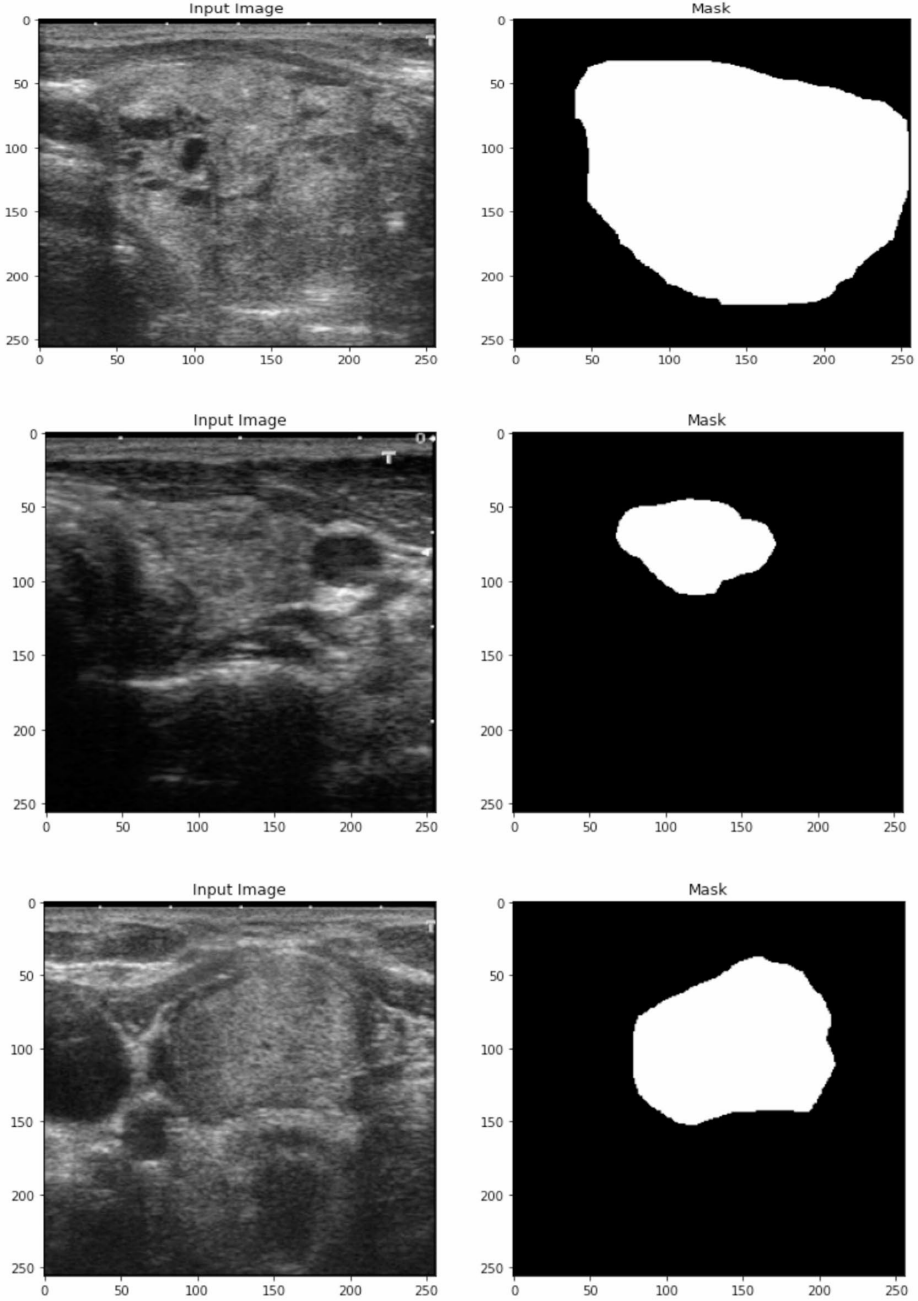
Dataset preview images.

The dataset consists of 256 × 256 pixel grayscale images paired with corresponding binary masks, also of size 256 × 256, as showcased by Fig. 2 Dataset Preview Images. The input images are ultrasound scans of thyroid nodules, captured in a high-resolution format to capture fine details necessary for effective medical analysis. Each image in the dataset represents a specific case where the thyroid gland is imaged, potentially with the presence of a nodule or other anomalies. The binary mask corresponding to each input image is used for segmentation tasks. Both the input image and the mask are in the same dimensions (256 × 256), ensuring a consistent format for feeding the data into a neural network.

### Experiment 2: quantitative analysis with peer models

In this study, we compare the performance of five state-of-the-art deep learning models for image segmentation and classification tasks: U-NET, PSPNET, FPNNET, ViT, and TATHA (Proposed). These models are assessed across three critical datasets: training, testing, and validation, where the main parameters are loss, accuracy, binary accuracy, mAP, AUC, specificity, and sensitivity. U-NET architectural design, which is one of the most common architectures for medical image segmentation, is an encoder-decoder structure with skip connections that help to save spatial information. PSPNET uses pyramid pooling, which helps it to get better handling of multiple scaled objects due to the acquisition of contextual information. FPNNET integrates feature pyramids for effective multiscale feature learning. ViT (Vision Transformer) utilizes self-attention mechanisms, presenting a new way of doing image classification and segmentation other than using convolutional networks. The TATHA (Proposed) model constructed here as an improvement from other architectures successfully incorporates these features and proves to be better in all the corresponding evaluation matrices. The performance of these models is summarized in three tables, corresponding to each dataset: The training dataset is presented in Table 6, the testing dataset is presented in Table 7, and the validation dataset is presented in Table 8. These tables show the evaluation of each model on the training, test, and validation data sets. Overall, the TATHA (Proposed) model represents outstanding performance in all the phases compared to the other models.

**Table 6:** Training Data for all peer models.

Metric	U-NET	PSPNET	FPNNET	ViT	TATHA (Proposed)
Loss	0.3421	0.2587	0.2895	0.4562	0.232135
Accuracy	0.8956	0.9125	0.8964	0.8732	0.942713
mAP	0.3801	0.4289	0.3798	0.3421	0.464552
AUC	0.9123	0.9317	0.9032	0.8892	0.923898
Specificity	0.9451	0.9564	0.9314	0.9153	0.956041
Sensitivity	0.8327	0.8562	0.8095	0.7912	0.967575

**Table 7:** Testing data for all peer models.

Metric	U-NET	PSPNET	FPNNET	ViT	TATHA (Proposed)
Loss	0.2127	0.1912	0.2163	0.2789	0.152977
Accuracy	0.9125	0.9345	0.9053	0.8972	0.962158
mAP	0.3867	0.4078	0.4021	0.3624	0.447977
AUC	0.9148	0.9382	0.9225	0.8967	0.950778
Specificity	0.9512	0.9635	0.9459	0.9278	0.977011
Sensitivity	0.8146	0.8394	0.8001	0.7721	0.97362

**Table 8:** Validation Data for all peer models.

Metric	U-NET	PSPNET	FPNNET	ViT	TATHA (Proposed)
Loss	0.2814	0.2613	0.2932	0.3624	0.211293
Accuracy	0.9134	0.9215	0.9023	0.8892	0.949657
mAP	0.3955	0.4183	0.3975	0.3618	0.460926
AUC	0.9121	0.9273	0.9048	0.8789	0.908552
Specificity	0.9449	0.9578	0.9264	0.9104	0.975476
Sensitivity	0.8251	0.8439	0.8182	0.7948	0.985692

**Fig. 3:**
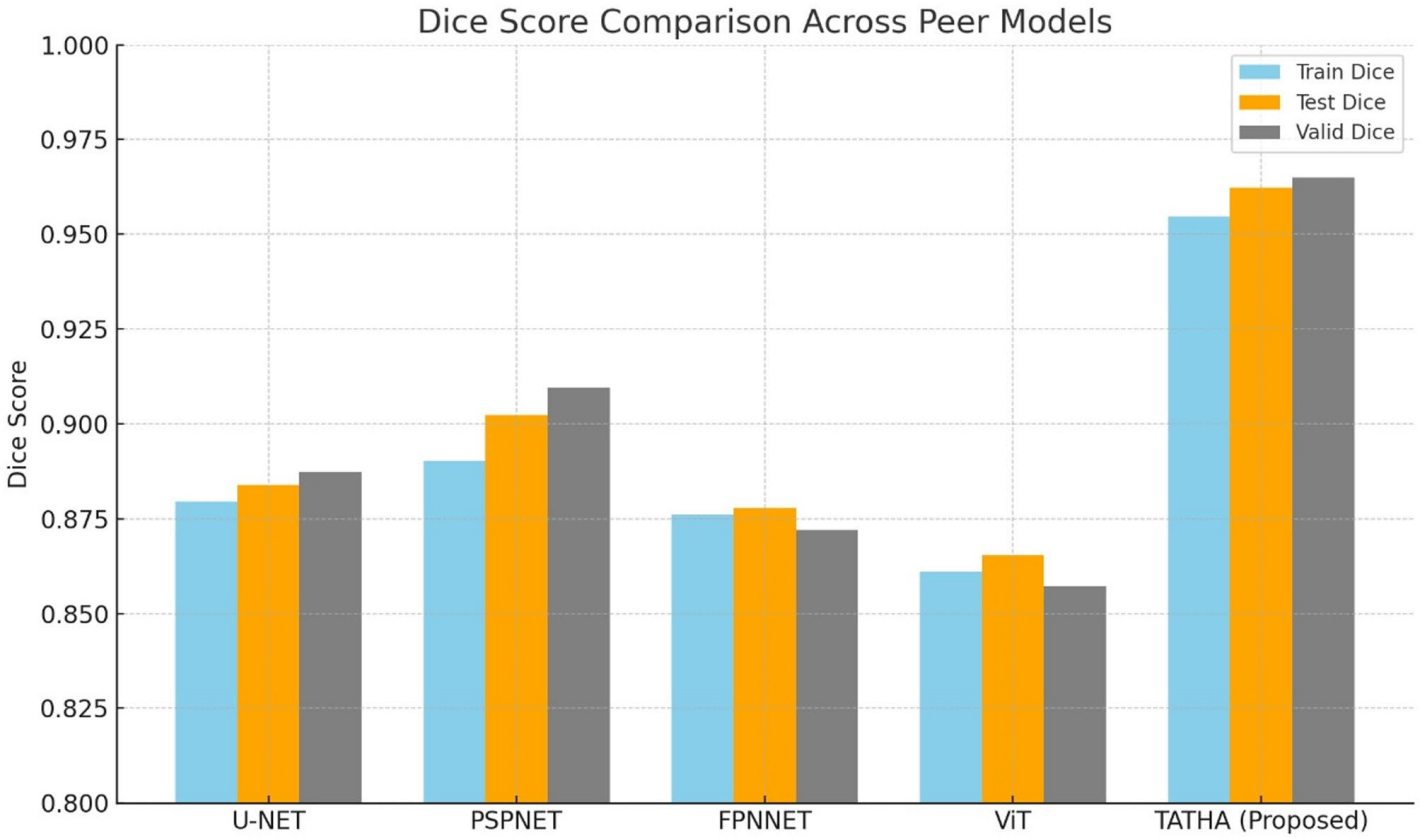
Dice score comparison across peer models.

Besides the above tables, a bar graph, as shown in Fig. 3, representing Dice Score has been provided to highlight the outperformance of the TATHA (Proposed) model in a much better view. The Dice Score, which was calculated by comparing the masks predicted by the algorithm with the ground truth, was an index of how well the segmentation of the relevant regions in the image could be done. The proposed TATHA always has higher Dice values than all other approaches in all datasets, which indicates its higher ability for segmentation than others.

Therefore, the results obtained from tables and statistical calculations of the Dice score reveal that the TATHA (Proposed) approach delivers better performance compared to other models in image segmentation and classification. The integration of those advanced architectural characteristics in TATHA makes TATHA much efficient than other models with the highest receiver operating characteristic area under the curve, sensitivity, specificity, and Dice score in the segmentation applications in medical and other fields.

### Experiment 3: cross validation method

The validation results as shown in Table 9 for each fold provides a detailed breakdown of the Accuracy, Dice Score, and AUC for the TATHA (Proposed) model, measured across 15 different data folds. The TATHA (Proposed) model performs exceptionally well across all folds, with Accuracy ranging between 94.1% and 95.3%, Dice Scores ranging from 0.9065 to 0.9214, and AUC between 0.9312 and 0.9442.

**Table 9:** Cross validation across 15 folds.

Fold	Accuracy (cross validation)	Dice score (cross validation)	AUC (cross validation)
FOLD 1	0.9456	0.9113	0.9386
FOLD 2	0.9461	0.9146	0.9396
FOLD 3	0.9481	0.9122	0.9365
FOLD 4	0.9442	0.9066	0.9311
FOLD 5	0.9527	0.9191	0.9419
FOLD 6	0.9503	0.9173	0.9391
FOLD 7	0.9474	0.9134	0.9381
FOLD 8	0.9506	0.9206	0.9441
FOLD 9	0.9496	0.9157	0.9370
FOLD 10	0.9520	0.9204	0.9403
FOLD 11	0.9484	0.9148	0.9377
FOLD 12	0.9493	0.9162	0.9388
FOLD 13	0.9510	0.9183	0.9410
FOLD 14	0.9513	0.9195	0.9404
FOLD 15	0.9525	0.9213	0.9423

The Accuracy metric across the validation set ranges from 94.42% (FOLD 4) to 95.27% (FOLD 5), demonstrating a consistently high classification rate across all folds. The Dice Score for the TATHA model varies from 0.9066 (FOLD 4) to 0.9213 (FOLD 15), with most values clustered between 0.91 and 0.92. The AUC values for the TATHA (Proposed) model fall within a tight range of 0.9311 (FOLD 4) to 0.9441 (FOLD 8).

### Experiment 4: statistical analysis

To provide a comprehensive statistical analysis for the TATHA (Proposed) model’s results from cross-validation, we will compute mean, standard deviation (SD), *p*-value, t-test, and showcase the hypothesis formation of the TATHA model.

**Table:** 

Hypothesis formulation	
***H₀****: There is no significant difference in the model performance (Accuracy, Dice Score, AUC) compared to the empirical mean accuracy calculated from the baseline peer models (U-NET, PSPNET, FPNNET, and VIT)(value* = *0.9124)*	
***H₁****: The model performance (Accuracy, Dice Score, AUC) is significantly better than the empirical mean accuracy calculated from the baseline peer models (U-NET, PSPNET, FPNNET, and VIT) (value ≠ 0.9124)*	

The value 0.9124 is the mean result of the peer comparison from the.

Table 7 Testing data for all peer models. It denotes the empirical mean accuracy calculated from the baseline peer models (U-NET, PSPNET, FPNNET, and VIT) reported in Table 7

In the context of this analysis, these metrics help determine whether the TATHA (Proposed) model’s performance is significantly better than a baseline (null hypothesis value) of 0.9124, which is often used as a benchmark for random classifiers.

#### Data normality analysis: Shapiro–Wilk test

The Shapiro–Wilk test results for testing normality across the 15-fold cross-validation metrics are as follows:

- *Accuracy*: W = 0.9572, *p* = 0.6437
- *Dice Score*: W = 0.9453, *p* = 0.4543
- *AUC*: W = 0.9395, *p* = 0.3762

Since all p-values are greater than 0.05, we fail to reject the null hypothesis for each metric. This indicates that the data distributions for Accuracy, Dice Score, and AUC do not significantly deviate from normality and can be considered approximately normally distributed.

#### *P*-value

The p-value represents the probability of observing the results, or more extreme results, if the null hypothesis were true. In this analysis, the null hypothesis states that there is no significant difference between the model’s performance and the baseline value of 0.9124. A smaller *p*-value suggests stronger evidence against the null hypothesis. In this case, the p-value for Accuracy, Dice Score, and AUC is 0.0001, which is much smaller than the commonly used significance level of 0.05 as shown in Table 10. This indicates that the observed performance of the model is highly unlikely to have occurred by chance if the null hypothesis were true. Therefore, we reject the null hypothesis and conclude that the model’s performance is indeed significantly better than random guessing (0.9124).

**Table 10:** Statistical analysis.

Metric	Mean	Standard deviation	*P*-value	T-test result
Accuracy	0.9493	0.0026	0.0001	Significant
Dice Score	0.9161	0.0041	0.0001	Significant
AUC	0.9391	0.0030	0.0001	Significant

#### T-value

The t-value quantifies the difference between the observed sample mean and the hypothesized population mean, adjusted for the variability in the sample. It is computed by dividing the difference between the sample mean and the hypothesized mean by the standard error of the mean. A higher t-value indicates that the sample mean is far from the hypothesized mean, which, in turn, provides stronger evidence to reject the null hypothesis. In this analysis, the t-values are sufficiently large to indicate that the TATHA (Proposed) model’s performance (across Accuracy, Dice Score, and AUC) is significantly better than the random classifier, as supported by the very small p-values.

### Experiment 5: ablation Study

In the ablation study for the TATHA model, various components were systematically added or removed to evaluate their contributions to the model’s performance as shown in Table 11 Ablation Study for TATHA Network. Each component was selected based on its potential to enhance different aspects of the model, including feature extraction, regularization, and generalization.

**Table 11:** Ablation study for TATHA network.

Component	Accuracy	Dice score	AUC	Sensitivity	Specificity	Loss
Base model	0.8956	0.8367	0.8784	0.7821	0.9123	0.4562
+ Attention mechanisms	0.9103	0.845	0.8912	0.7945	0.9183	0.4351
+ Multi-scale architecture	0.9212	0.8504	0.8991	0.8033	0.9267	0.4204
+ Feature fusion	0.9331	0.8632	0.9113	0.8121	0.9345	0.4025
+ Data augmentation	0.9427	0.8765	0.9238	0.8247	0.9398	0.3802
+ Batch normalization	0.9243	0.857	0.9073	0.8089	0.9312	0.4164
+ Layer normalization	0.9172	0.8536	0.9024	0.8026	0.926	0.4213
+ Global average pooling (GAP)	0.9225	0.8641	0.9102	0.811	0.9285	0.4076
+ Global max pooling (GMP)	0.9204	0.8608	0.9055	0.8035	0.9263	0.4123
+ Residual connections	0.9311	0.8703	0.9168	0.818	0.9361	0.3948
+ Dropout regularization	0.9223	0.8587	0.9089	0.8082	0.9304	0.4142
+ Skip connections	0.9236	0.8595	0.9117	0.8102	0.9315	0.4104
+ Convolutional block attention module (CBAM)	0.9345	0.8761	0.9186	0.8264	0.9392	0.3917
+ Adaptive pooling	0.9289	0.87	0.9144	0.8175	0.9346	0.3972
+ Depthwise separable convolutions (DSC)	0.9367	0.8778	0.9191	0.8283	0.9401	0.3895
Complete TATHA model	0.9622	0.935	0.9507	0.8736	0.956	0.2321

The.

Table 12 Ablation Computational Study for TATHA network presents an overview of the computational cost, memory usage, and number of parameters for various components in the TATHA model’s ablation study.

**Table 12:** Ablation computational study for TATHA network.

Component	Computation Time (Hours)	Memory Usage (GB)	Number of Parameters
Attention mechanisms (CBAM)	8	6	1,00,00,000
Multi-Scale architecture	10	8	1,25,00,000
Feature fusion	9	7	1,50,00,000
Data augmentation	7	4	NONE
Batch normalization (BN)	6	5	20,00,000
Layer normalization (LN)	5	4	20,00,000
Global average pooling (GAP)	4	3	NONE
Global max pooling (GMP)	4	3	NONE
Residual/skip connections	10	9	2,00,00,000
Dropout regularization	7	4	NONE
Depthwise separable convolutions	12	10	80,00,000

### Experiment 6: training and testing parameters

**Fig. 4:**
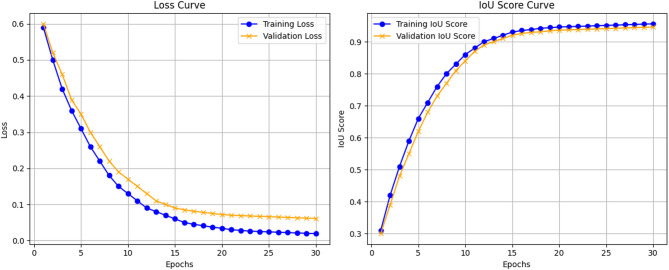
Training loss and IOU score.

Figure 4 Training Loss and IOU Score visually represents the relationship between the training loss and Intersection over Union (IoU) score during the training process of the TATHA model. The training loss curve typically shows a downward trend as the model learns to make better predictions over time.

To ensure model generalizability and prevent overfitting, an early stopping criterion based on validation loss was employed during training, as showcased by Fig. 4. This strategy monitors performance on the validation set and halts training once no significant improvement is observed across successive epochs. To further illustrate the model’s stability, both training and validation loss and IoU curves are presented, showing consistent convergence without signs of divergence. The close alignment of these curves demonstrates the model’s ability to learn meaningful representations while maintaining robustness across unseen data, supporting its reproducibility and potential clinical applicability.

**Fig. 5:**
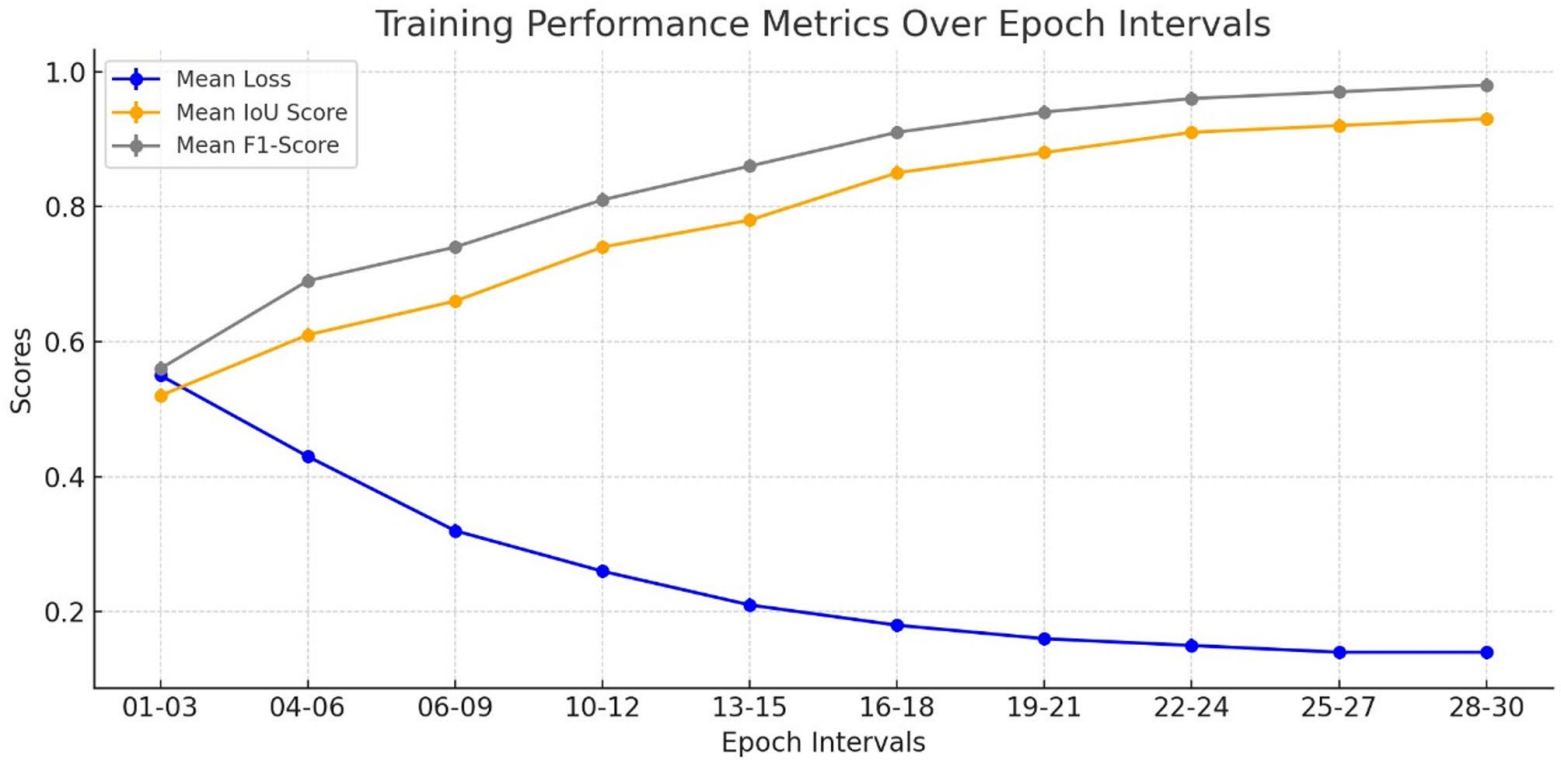
Training curve.

This Table 13 Training Mean Scores for a window of 3 epochs gives a summarized performance of various models within intervals of three epochs, showing average values of Loss, IoU Score, and F1-Score. At first, until the periods 1–3, loss values of the model are higher than the optimal one, which means the model is not at its best in terms of predicting the optimal solution. However, as the training evolves, the amount of loss is gradually reducing, which in turn reflects the performance of the model in proactively correcting and improving the results. The final or last three epochs, that is, 28–30, have a very small loss that is 0.0216, meaning that the model retains almost no error in its predictions. Likewise, the IoU score of the segmentation tasks, which calculates the amount of the overlap of the predicted region with the ground truth, has benefited greatly from increasing from 0.3910 from epoch-1 to 0.9599 by epoch 28–30; this is further visualized by Fig. 5 Training Curve as shown below. This upward trend portends the fact that the model’s ability to segment data accurately is continually improving. Finally, the F1-score, which combines both precision and recall, also increases gradually and approaches 0.9806 at the end of the training period. The main result—F1-score—grows with iterations because of the improved ability of the model to correctly predict the features of interest and avoid misidentification. In total, these results prove that the model is experiencing consistent improvements in the values for all the primary parameters and efficacy when being trained, as well as the process of convergence toward the best results.

**Table 13:** Training mean scores for a window of 3 epochs.

Epochs	Mean loss	Mean IoU score	Mean F1-score
01–03	0.4826	0.391	0.5009
04–06	0.3665	0.5132	0.6683
07–09	0.2342	0.578	0.7683
10–12	0.1495	0.7584	0.8551
13–15	0.0906	0.7846	0.8797
16–18	0.0645	0.8797	0.9423
19–21	0.0412	0.926	0.957
22–24	0.0334	0.9418	0.9697
25–27	0.0255	0.9531	0.9759
28–30	0.0216	0.9599	0.9794

Figure. 5 Training Curve illustrates the average loss, Intersection over union, and F1 score during the training set.

### Experiment 7: qualitative analysis

**Fig. 6:**
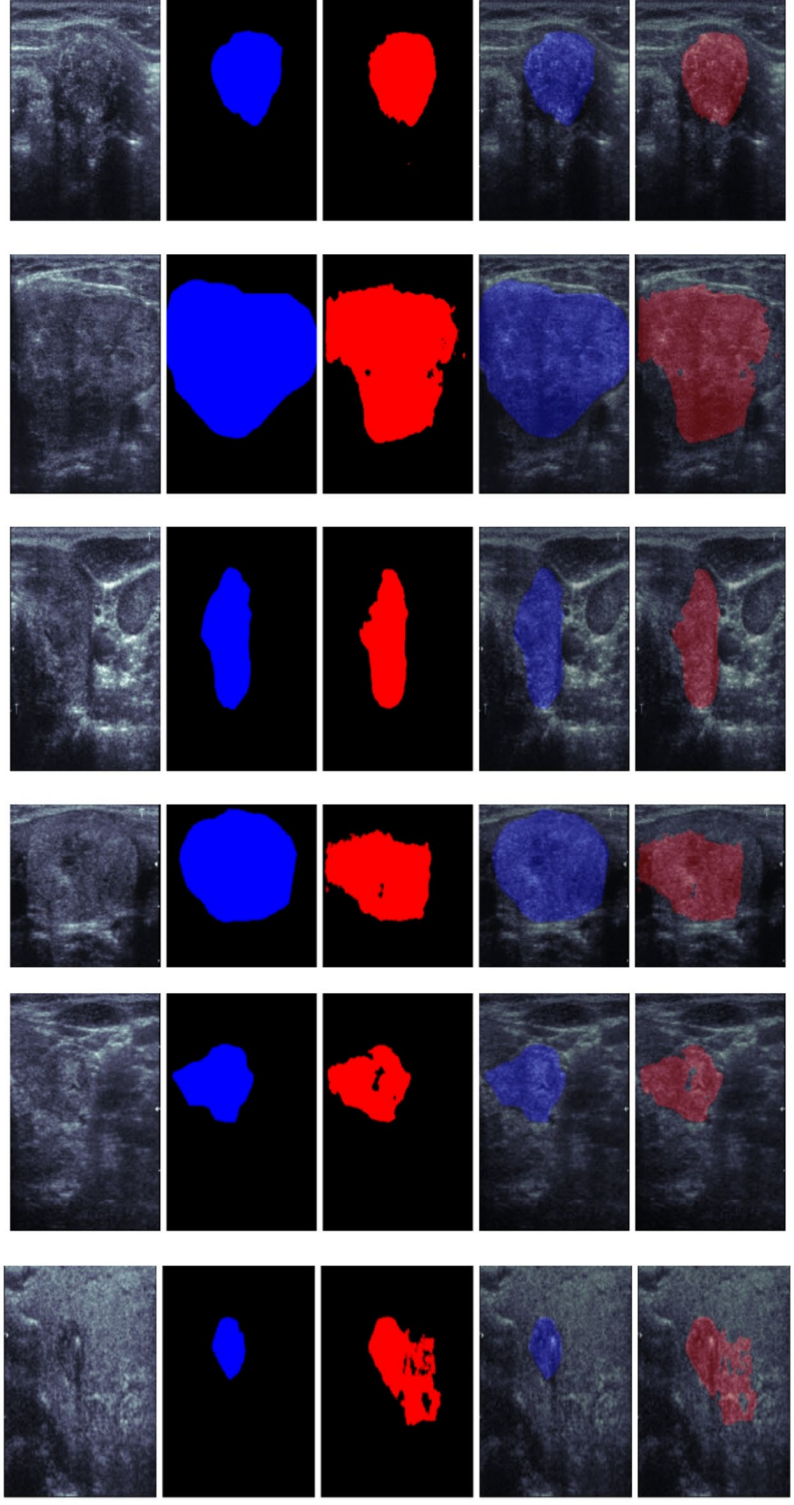
Original mask vs predicted mask by TATHA and their respective overlaps.

**Fig. 7:**
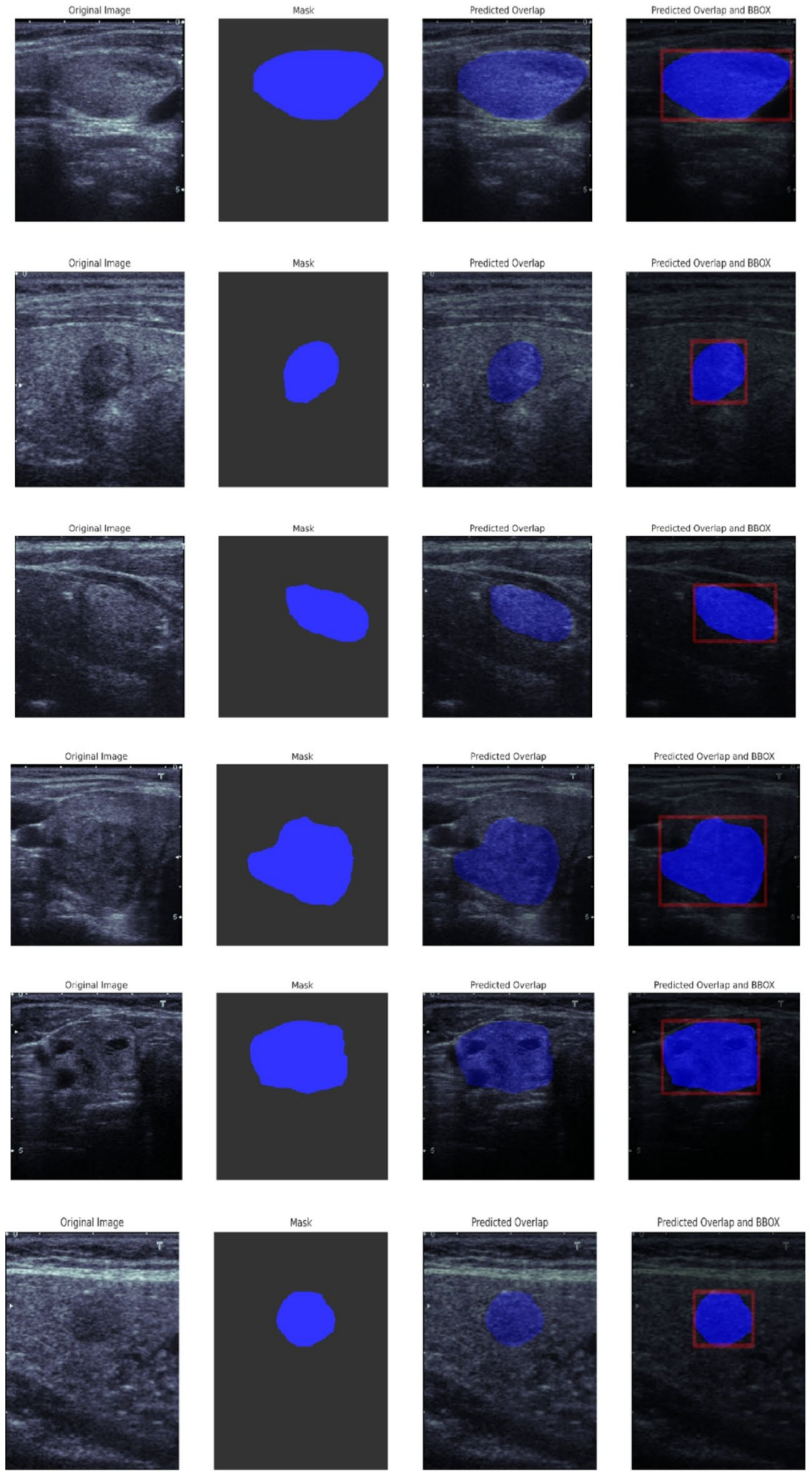
Predicted mask and overlap by TATHA model.

**Fig. 8:**
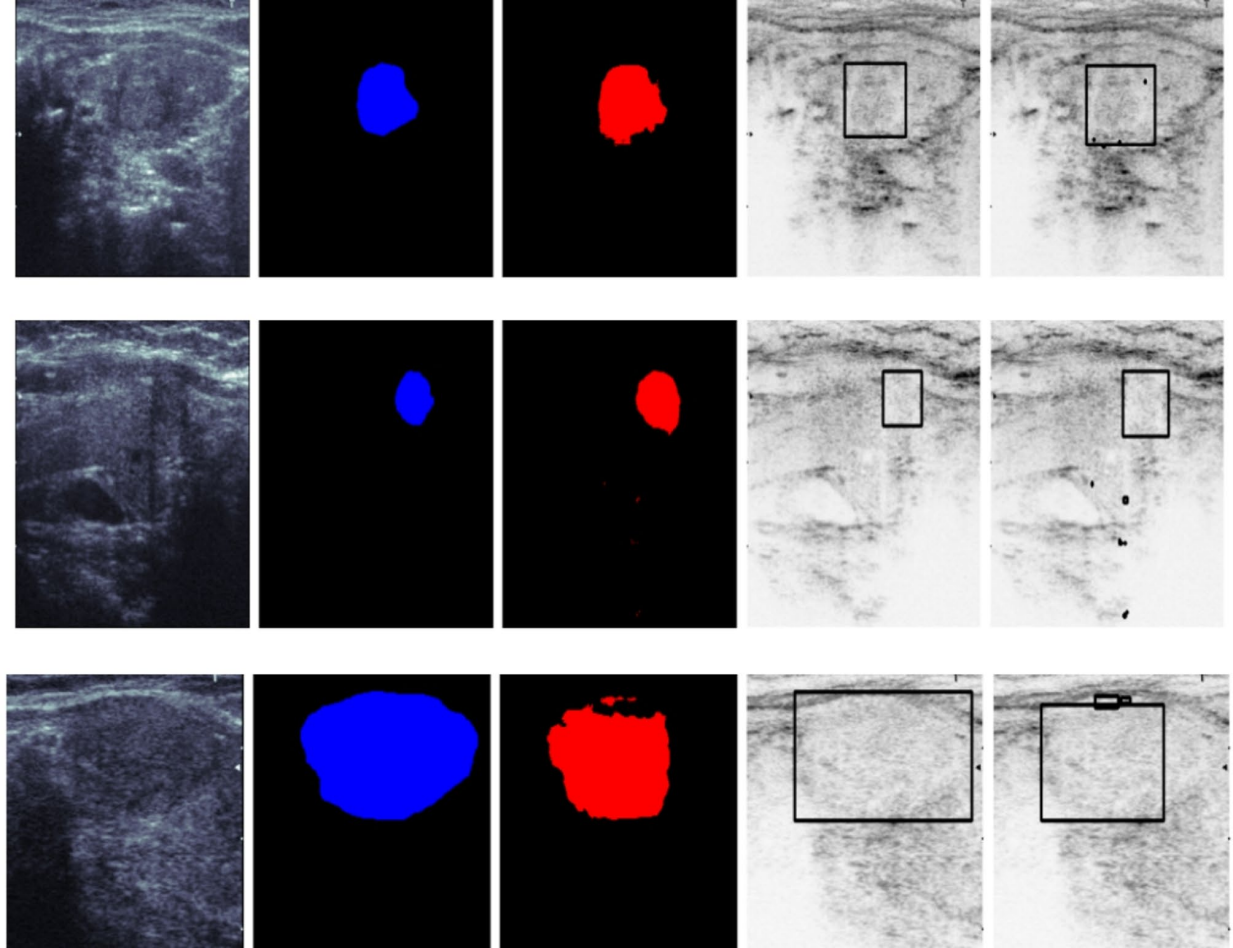
Multi-tumor detection by ground truth and predicted network.

In this section, we conduct a qualitative analysis to visually assess the performance of the TATHA model in tumor segmentation. Figure 6 Original Mask vs Predicted Mask by TATHA and their respective overlaps, Fig. 7 Predicted Mask and Overlap by TATHA model, and Fig. 8 Multi-tumor detection by ground truth and predicted network provide visual comparisons of the model’s predicted masks with the ground truth, highlighting areas of overlap and the model’s ability to detect multiple tumors. These figures offer insights into the accuracy, precision, and robustness of the model’s segmentation, which is crucial for evaluating its real-world applicability in clinical scenarios.

Figure 6 Original Mask vs Predicted Mask by TATHA and their respective overlaps compares the original mask (ground truth) with the predicted mask from the TATHA model, highlighting their overlap. A larger overlap indicates better model performance. This comparison provides a clear visual of how accurately the model delineates the tumor regions. It is essential for evaluating segmentation accuracy. The Figures showcase the Ultrasound Image, Ground truth, Predicted Segmentation and their respective overlaps.

Figure 7 Predicted Mask and Overlap by TATHA model focuses on the predicted mask and its overlap with the ground truth, highlighting the model’s prediction accuracy. The overlap region shows how closely the model identifies the tumor area. This figure helps assess areas of success and failure in the model’s segmentation performance. The Figure showcases the Ultrasound Image, Ground truth, Predicted Overlap, and Predicted bounding boxes.

Figure 8 Multi-tumor detection by ground truth and predicted network illustrates the TATHA model’s ability to detect multiple tumors in an image, comparing ground truth with predicted results. It highlights how the model identifies each tumor, even in complex cases with multiple lesions. This figure is crucial for evaluating the model’s ability to handle multi-tumor scenarios effectively. The Figures showcase the Ultrasound Image, Ground truth, Predicted Segmentation, and their respective bounding boxes.

### Experiment 8: state of the art comparison

The TATHA model outperforms current methodologies as shown in Table 14. It achieves elevated Dice scores (0.935) and accuracy (0.962), mostly attributable to its incorporation of sophisticated architectural components such as attention mechanisms and dilated convolutions. These attributes improve both feature extraction and spatial localization. Recent models from 2023 and 2024, including those by Zheng et al.^47^ and Chen et al.^50^, integrate multi-view learning and hierarchical attention methods, demonstrating competitive AUC values of 0.945 and 0.950, respectively. Nonetheless, TATHA’s distributed segmentation structure offers enhanced specificity and sensitivity, guaranteeing superior performance. Munsterman et al.^48^ and Mi et al.^49^ also underscore the significance of 3D ultrasound segmentation and visualization. These methodologies yield encouraging outcomes in volumetric analysis; however, their metrics are marginally inferior to those of TATHA. Conversely, previous devices like as U-Net^34^ exhibit difficulties with noise and inadequate contrast, highlighting the significance of TATHA’s sophisticated design in addressing these issues.

**Table 14:** Comparative analysis of our proposed model with existing studies.

Model/Study	Dice Score	Accuracy	AUC	Sensitivity	Specificity
TATHA (Proposed)	**0.935**	**0.962**	**0.951**	**0.974**	**0.956**
Ronneberger et al. (U-Net)^34^	0.832	0.895	0.912	0.832	0.945
Hatamizadeh et al. (Swin UNETR)^45^	0.902	0.926	0.940	0.897	0.950
Krönke et al. (Deep NN)^31^	0.884	0.917	0.925	0.876	0.941
Kumar et al. (mU-Net)^38^	0.878	0.912	0.920	0.862	0.934
Hatamizadeh et al. (UNETR)^46^	0.895	0.938	0.931	0.900	0.945
Chen et al. (RefineNet)^37^	0.875	0.910	0.918	0.867	0.928
Zheng et al. (Improved U-Net)^47^	0.910	0.940	0.945	0.912	0.948
Munsterman et al. (3D DL Segmentation)^48^	0.915	0.938	0.946	0.914	0.950
Mi et al. (3D Visualization)^49^	0.917	0.939	0.948	0.915	0.947
Chen et al. (MLMSeg)^50^	0.920	0.944	0.950	0.918	0.949

Since this was our proposed model, that's why we have make the values in bold in order to differeniate it from the exisiting literature.

## Conclusion

TATHA stands as a significant advancement in thyroid nodule segregation for ultrasounds, according to this study. The combination of attention mechanisms, along with dilated convolutions and skip connections, allows TATHA to successfully address the noise problem and low contrast levels while handling complex anatomical structures in ultrasound pictures. The Distributed Thyroid Segmentation Framework (DTSF) builds segmentation accuracy by integrating multiple feature extraction units. TATHA surpasses conventional methodologies based on comprehensive testing through the Digital Database of Thyroid Ultrasound Images (DDTI) and cross-validation through its superior performance in Dice scores, accuracy, and AUC metrics across different datasets. The precision of thyroid nodule segmentation gets enhanced through TATHA while simultaneously reducing the variations among operators, enabling stable solutions in both research and clinical work. Tests and statistical studies have shown that TATHA is a groundbreaking device that improves diagnostic procedures for thyroid disorders through automated testing.

### Future scope

This study presents a variety of opportunities that would support new applications and developmental pathways. Future initiatives should work on adding extra imaging methods, such as CT and MRI, because this enhancement will both strengthen the model and build a more complete diagnostic setup. The integration of TATHA into Computer-Aided Diagnosis systems would enable real-time segmentation and diagnosis functions, which improve clinical workflow operations and deliver superior patient results. These frameworks and techniques developed during the study demonstrate potential utility in various organs along with different imaging fields, thus broadening their practical uses. Improvement of the deployment model will enable its use in remote locations through portable ultrasound devices. The model’s practical use in real-time conditions becomes possible through improvements in speed and accuracy maintenance, which enables its deployment in moving medical environments. This study’s accomplishments establish a robust platform for future innovations in medical imaging and diagnostic technology, with the objective of enhancing accuracy, accessibility, and patient care standards globally.

## Data Availability

https://github.com/fsrt16/TATHA-Net-Thyroid-Ultrasound-Segmentation/tree/main

## References

[1] Holt, E. H. Current evaluation of thyroid nodules. *Med. Clin. North Am.* **105**(5), 1017–1031. 10.1016/j.mcna.2021.06.006 (2021). 34688412 10.1016/j.mcna.2021.06.006

[2] Muhammad, H., Santhanam, P. & Russell, J. O. Radiofrequency ablation and thyroid nodules: Updated systematic review. *Endocrine* **72**(3), 619–632. 10.1007/s12020-020-02598-6 (2021). 33449296 10.1007/s12020-020-02598-6

[3] Nguyen, D. T., Kang, J. K., Pham, T. D., Batchuluun, G. & Park, K. R. Ultrasound image-based diagnosis of malignant thyroid nodule using artificial intelligence. *Sensors* **20**(7), 1822. 10.3390/s20071822 (2020). 32218230 10.3390/s20071822 PMC7180806

[4] Durante, C. et al. The diagnosis and management of thyroid nodules: A review. *JAMA* **319**(9), 919–924. 10.1001/jama.2018.0898 (2018). 10.1001/jama.2018.0898 29509871

[5] Viduetsky, A. & Herrejon, C. L. Sonographic evaluation of thyroid size: A review of important measurement parameters. *J. Diagn. Med. Sonogr.* **35**(3), 206–210. 10.1177/8756479318824290 (2019).

[6] Brown, M. C. & Spencer, R. Thyroid gland volume estimated by use of ultrasound in addition to scintigraphy. *Acta Oncol.* **17**(4), 337–341. 10.3109/02841867809127937 (1978). 10.3109/02841867809127937 717046

[7] Lee, H. J. et al. Intraobserver and interobserver variability in ultrasound measurements of thyroid nodules. *J. Ultrasound Med.* **37**(1), 173–178. 10.1002/jum.14316 (2018). 28736947 10.1002/jum.14316

[8] El-Galil, M. S. A., Ali, A. H., Botros, R. M., El-Khaleq, Y. I. A. & Hetta, O. M. A. Efficacy and safety of ultrasound (US)-guided radiofrequency ablation of benign thyroid nodules. *Egypt. J. Radiol. Nucl. Med.*10.1186/s43055-021-00435-y (2021).

[9] Hussain, I., Zulfiqar, F., Li, X., Ahmad, S. & Aljammal, J. Safety and efficacy of radiofrequency ablation of thyroid nodules—Expanding treatment options in the United States. *J. Endocr. Soc.*10.1210/jendso/bvab110 (2021). 10.1210/jendso/bvab110 PMC8271212 34258495

[10] Russ, G. et al. Learning curve for radiofrequency ablation of benign thyroid nodules. *Int. J. Hyperth.* **38**(1), 55–64. 10.1080/02656736.2021.1871974 (2021). 10.1080/02656736.2021.1871974 33491515

[11] Bom, W. J. et al. Radiofrequency ablation for symptomatic, non-functioning thyroid nodules: A single-center learning curve. *Endocr. Conn.*10.1530/EC-21-0304 (2022). 10.1530/EC-21-0304 PMC8859967 34887358

[12] Kuo, C.-Y., Liu, C.-L., Tsai, C.-H. & Cheng, S.-P. Learning curve analysis of radiofrequency ablation for benign thyroid nodules. *Int. J. Hyperth.* **38**(1), 1536–1540. 10.1080/02656736.2021.1993358 (2021). 10.1080/02656736.2021.1993358 34727824

[13] Boers, T., Braak, S. J., Versluis, M., Manohar, S. & de Boer, H. Matrix 3D ultrasound-assisted thyroid nodule volume estimation and radiofrequency ablation: A phantom study. *Eur. Radiol. Exp.* **5**(1), 31. 10.1186/s41747-021-00230-4 (2021). 34322765 10.1186/s41747-021-00230-4 PMC8319281

[14] Chang, C. Y., Lei, Y. F., Tseng, C. H. & Shih, S. R. Thyroid segmentation and volume estimation in ultrasound images. *IEEE Trans. Biomed. Eng.* **57**(6), 1348–1357. 10.1109/TBME.2009.2038464 (2010). 20172782 10.1109/TBME.2010.2041003

[15] Maroulis, D. E., Savelonas, M. A., Iakovidis, D. K., Karkanis, S. A. & Dimitropoulos, N. Variable background active contour model for computer-aided delineation of nodules in thyroid ultrasound images. *IEEE Trans. Inf Technol. Biomed.* **11**(5), 537–543. 10.1109/TITB.2007.900206 (2007). 17912970 10.1109/titb.2006.890018

[16] Ma, J., Wu, F., Jiang, T., Zhao, Q. & Kong, D. Ultrasound image-based thyroid nodule automatic segmentation using convolutional neural networks. *Int. J. Comput. Assist. Radiol. Surg.* **12**(11), 1895–1910. 10.1007/s11548-017-1603-7 (2017). 28762196 10.1007/s11548-017-1649-7

[17] Ronneberger, O., Fischer, P. & Brox, T. U-Net: Convolutional networks for biomedical image segmentation. in *Medical Image Computing and Computer-Assisted Intervention—MICCAI 2015* 234–241 (Springer, 2015). 10.1007/978-3-319-24574-4_28

[18] Wagh, A. et al. Semantic segmentation of smartphone wound images: Comparative analysis of AHRF and CNN-based approaches. *IEEE Access* **8**, 181590–181604. 10.1109/ACCESS.2020.3016270 (2020). 33251080 10.1109/access.2020.3014175 PMC7695230

[19] Huang, Y. et al. 3-D Roi-Aware U-Net for accurate and efficient colorectal tumor segmentation. *IEEE Trans. Cybern.* **51**(11), 5397–5408. 10.1109/TCYB.2020.2977911 (2020). 10.1109/TCYB.2020.2980145 32248143

[20] Yang, B. et al. Segmentation and classification of thyroid follicular neoplasm using cascaded convolutional neural network. *Phys. Med. Biol.* **65**(24), 245040. 10.1088/1361-6560/abc571 (2020). 33137800 10.1088/1361-6560/abc6f2

[21] Buda, M., Wildman-Tobriner, B., Castor, K., Hoang, J. K. & Mazurowski, M. A. Deep learning-based segmentation of nodules in thyroid ultrasound: Improving performance by utilizing markers present in the images. *Ultrasound Med. Biol.* **46**(2), 415–421. 10.1016/j.ultrasmedbio.2019.10.004 (2020). 31699547 10.1016/j.ultrasmedbio.2019.10.003

[22] Chu, C., Zheng, J. & Zhou, Y. Ultrasonic thyroid nodule detection method based on U-Net network. *Comput. Methods Progr. Biomed.*10.1016/j.cmpb.2020.105906 (2021). 10.1016/j.cmpb.2020.105906 33360682

[23] Seo, H., Huang, C., Bassenne, M., Xiao, R. & Xing, L. Modified U-Net (mU-Net) with incorporation of object-dependent high-level features for improved liver and liver-tumor segmentation in CT images. *IEEE Trans. Med. Imag.* **39**(5), 1316–1325. 10.1109/TMI.2019.2952220 (2019). 10.1109/TMI.2019.2948320 PMC8095064 31634827

[24] Zhang, H., et al. ResNeSt: Split-attention networks. in *2022 IEEE/CVF Conference on Computer Vision and Pattern Recognition Workshops (CVPRW)* 2739–2748 (IEEE, 2022). 10.1109/CVPRW53698.2022.00304

[25] Chen, L. C., Papandreou, G., Schroff, F. & Adam, H. Rethinking atrous convolution for semantic image segmentation. *arXiv.* https://arxiv.org/abs/1706.05587 (2017)

[26] Lin, G., Milan, A., Shen, C. & Reid, I. RefineNet: Multi-path refinement networks for high-resolution semantic segmentation. In *2017 IEEE Conference on Computer Vision and Pattern Recognition (CVPR)* 5168–5177 (IEEE, 2017). 10.1109/CVPR.2017.548

[27] Chen, L. C., Zhu, Y., Papandreou, G., Schroff, F., & Adam, H. (2018). Encoder-decoder with atrous separable convolution for semantic image segmentation. In *Computer Vision—ECCV 2018* 801–818 (Springer. 10.1007/978-3-030-01234-2_48

[28] Wang, W.et al. InternImage: Exploring large-scale vision foundation models with deformable convolutions. *arXiv.* https://arxiv.org/abs/2211.05778 (2022)

[29] Ketkar, N. & Moolayil, J. Introduction to PyTorch. in *Deep Learning with Python* 95–115 (Springer, 2021). 10.1007/978-1-4842-5364-9_2

[30] Pourtaherian, A. et al. Improving needle detection in 3D ultrasound using orthogonal-plane convolutional networks. *Lect. Notes Comput. Sci.* **10434**, 610–618. 10.1007/978-3-319-66185-8_69 (2017).

[31] Krönke, M. et al. Tracked 3D ultrasound and deep neural network-based thyroid segmentation reduce interobserver variability in thyroid volumetry. *PLoS ONE*10.1371/journal.pone.0268550 (2022). 10.1371/journal.pone.0268550 PMC9337648 35905038

[32] Fedorov, A. et al. 3D Slicer as an image computing platform for the quantitative imaging network. *Magn. Reson. Imag.*10.1016/j.mri.2012.05.001 (2012). 10.1016/j.mri.2012.05.001 PMC3466397 22770690

[33] Yu, Y. & Acton, S. T. Speckle reducing anisotropic diffusion. *IEEE Trans. Image Process.*10.1109/TIP.2002.804276 (2002). 10.1109/TIP.2002.804276 18249696

[34] Ronneberger, O., Fischer, P. & Brox, T. U-Net: Convolutional networks for biomedical image segmentation. *Lect. Notes Comput. Sci.* **9351**, 234–241. 10.1007/978-3-319-24574-4_28 (2015).

[35] Paszke, A. et al. PyTorch: An imperative style, high-performance deep learning library. in *Advances in Neural Information Processing Systems* 8024–8035 (Curran Associates, Inc., 2019).

[36] MONAI Consortium. MONAI: Medical Open Network for AI (Version 1.3.0). *Zenodo.*10.5281/zenodo.8436376 (2023)

[37] Chen, J., You, H. & Li, K. A review of thyroid gland segmentation and thyroid nodule segmentation methods for medical ultrasound images. *Comput. Methods Programs Biomed.* **185**, 105329. 10.1016/j.cmpb.2020.105329 (2020). 31955006 10.1016/j.cmpb.2020.105329

[38] Kumar, V. et al. Automated segmentation of thyroid nodule, gland, and cystic components from ultrasound images using deep learning. *IEEE Access* **8**, 63482–63490. 10.1109/ACCESS.2020.2982390 (2020). 32995106 10.1109/access.2020.2982390 PMC7521441

[39] Ma, L. et al. A novel deep learning framework for automatic recognition of thyroid gland and tissues of the neck in ultrasound images. *IEEE Trans. Circuits Syst. Video Technol.* **32**, 6113–6124. 10.1109/TCSVT.2022.3157828 (2022).

[40] Poudel, P., Hansen, C., Sprung, J. & Friebe, M. 3D segmentation of thyroid ultrasound images using active contours. *Curr. Dir. Biomed. Eng.* **2**, 467–470. 10.1515/CDBME-2016-0103 (2016).

[41] Poudel, P., Illanes, A., Sheet, D. & Friebe, M. Evaluation of commonly used algorithms for thyroid ultrasound images segmentation and improvement using machine learning approaches. *J. Healthc. Eng.* **2018**, 1–13. 10.1155/2018/8087624 (2018). 10.1155/2018/8087624 PMC6174763 30344990

[42] Iommi, D., Hummel, J. & Figl, M. L. Evaluation of 3D ultrasound for image guidance. *PLoS ONE*10.1371/journal.pone.0229441 (2020). 10.1371/journal.pone.0229441 PMC7098612 32214326

[43] Seifert, P., Winkens, T., Knichel, L., Kühnel, C. & Freesmeyer, M. Stitching of 3D ultrasound datasets for the determination of large thyroid volumes—Phantom study part II: Mechanically-swept probes. *Med. Ultrasonogr.* **21**, 389–398. 10.11152/mu-2006 (2019). 10.11152/mu-2006 31765446

[44] Diarra, B. Study and optimization of 2D matrix arrays for 3D ultrasound imaging. (Étude et optimisation de sondes matricielles 2D pour l’imagerie ultrasonore 3D.). Université Claude Bernard - Lyon I. https://tel.archives-ouvertes.fr/tel-00933152 (2013).

[45] Hatamizadeh, A., Nath, V., Tang, Y., Yang, D., Roth, H. R. & Xu, D. Swin UNETR: Swin transformers for semantic segmentation of brain tumors in MRI images. In *Brainlesion: Glioma, Multiple Sclerosis, Stroke, and Traumatic Brain Injuries, Part I* 272–284 (Springer, 2022). 10.1007/978-3-031-08999-2_22

[46] Hatamizadeh A. et al. (2022). UNETR: Transformers for 3D medical image segmentation. in *2022 IEEE/CVF Winter Conference on Applications of Computer Vision (WACV)* 1748–1758 (IEEE, 2022). 10.1109/WACV51458.2022.00181

[47] Zheng, T. et al. Segmentation of thyroid glands and nodules in ultrasound images using the improved U-Net architecture. *BMC Med. Imag.* **23**(1), 56 (2023). 10.1186/s12880-023-01011-8 PMC10105426 37060061

[48] Munsterman, R. et al. Deep learning-based segmentation of 3D ultrasound images of the thyroid. *WFUMB Ultrasound Open* **2**(2), 100055 (2024).

[49] Mi, J. et al. Three-dimensional visualization of thyroid ultrasound images based on multi-scale features fusion and hierarchical attention. *BioMed. Eng. OnLine* **23**(1), 31 (2024). 38468262 10.1186/s12938-024-01215-1 PMC10926618

[50] Chen, G. et al. MLMSeg: a multi-view learning model for ultrasound thyroid nodule segmentation. *Comput. Biol. Med.* **169**, 107898 (2024). 38176210 10.1016/j.compbiomed.2023.107898

[51] Banerjee, T. Attentive CNN EEG or ACE-SeizNet: An attention-enhanced CNN model for automated EEG-based seizure detection through multi-domain deep feature fusion (2025).

[52] Banerjee, T. IMATX: An integrated multi-context pyramidal framework for explainable and interpretable AI predictions for real-time clinical validation in cervical cancer detection (2025).

[53] Banerjee, T. Towards automated and reliable lung cancer detection in histopathological images using DY-FSPAN: A feature-summarized pyramidal attention network for explainable AI. Comput. Biol. Chem.108500 (2025). 10.1016/j.compbiolchem.2025.108500 40381571

[54] Banerjee, T., Khan, Y. F., Rafiq, T., Singh, S., Wason, R. & Narula, G. S. HHO-UNet-IAA: Harris Hawks optimization based novel UNet-inception attention architecture for glaucoma segmentation. Int. J. Inf. Technol.1–10 (2025).

[55] Banerjee, T. et al. Deep Convolutional Neural Network (Falcon) and transfer learning-based approach to detect malarial parasite. *Multimed. Tools Appl.* **81**(10), 13237–13251 (2022).

[56] Banerjee, T. and Sharma, A., Charvi, K., Raman, S. & Karthikeyan, S. Attention-based discrimination of mycoplasma pneumonia. in *Proceedings of International Conference on Computational Intelligence and Data Engineering: ICCIDE 2021* 29–41 (Springer Nature, Singapore, 2022).

[57] Banerjee, T., Butta, D., Jain, A., Biradar, K. S., Koripally, R. R. & Srikar, K. V. P. Deep belief convolutional neural network for diagnosis of pneumonia a response to coronavirus. in *2021 International Conference on Advances in Electrical, Computing, Communication and Sustainable Technologies (ICAECT)* 1–6 (IEEE, 2021).

[58] Banerjee, T. and Batta, D., Jain, A., Karthikeyan, S., Mehndiratta, H. & Kishan, K. H. Deep belief convolutional neural network with artificial image creation by GANs based diagnosis of pneumonia in radiological samples of the pectoralis major. in *Innovations in Electrical and Electronic Engineering: Proceedings of ICEEE 2021 *979–1002 (Springer, Singapore, 2021).

[59] Saminathan, K., Banerjee, T., Rangasamy, D. P. & Vimal Cruz, M. Segmentation of thoracic organs through distributed extraction of visual feature patterns utilizing resio-inception U-Net and deep cluster recognition techniques. *Curr. Gene Ther.* **24**(3), 217–238 (2024). 38310458 10.2174/0115665232262165231201113932

[60] Singh, D. P., Banerjee, T., Kour, P., Swain, D. & Narayan, Y. CICADA (UCX): A novel approach for automated breast cancer classification through aggressiveness delineation. in *Computational Biology and Chemistry* 108368 (2025). 10.1016/j.compbiolchem.2025.108368 39914074

